# Administration of melatonin nanoparticles improves testicular blood flow, echotexture of testicular parenchyma, scrotal circumference, and levels of estradiol and nitric oxide in prepubertal ossimi rams under summer heat stress

**DOI:** 10.1007/s11259-024-10563-1

**Published:** 2024-10-23

**Authors:** Eman Fayez, Haney Samir, Fady Sayed Youssef, Ali Salama, Mohamed AI ElSayed

**Affiliations:** 1 Department of Theriogenology, Faculty of Veterinary Medicine, Cairo University, Giza, 12211 Egypt; 2 Pharmacology Department, Faculty of Veterinary Medicine, Cairo University, Giza, Egypt

**Keywords:** Color Doppler ultrasonography, Estradiol, Nano melatonin, Nitric oxide, Rams, Testicular echotexture, Testicular vascularization, Total antioxidant capacity

## Abstract

Environmental heat stress (HS) impairs reproductive efficiency in farm animals. This study investigated, for the first time, how the melatonin and melatonin nanoparticles treatment affected the testicular hemodynamics, testicular volume, echotexture [Pixel intensity (PIX) and integrated density (IND)], scrotal circumference, serum concentration of testosterone (T), estradiol (E_2_), nitric oxide (NO), and total antioxidant capacity (TAC) in prepubertal Ossimi ram lambs in hot climatic conditions. The lambs undergoing examination had a temperature humidity index (THI) of 87.05 ± 1.70, indicating severe HS condition. Fifteen prepubertal Ossimi ram lambs were exposed to a single s.c injection of either nano melatonin (nano melatonin group; 20 mg/ram; n _=_ 5) or melatonin suspended in two ml of corn oil (melatonin group; 40 mg/ram; n _=_ 5) or two ml of corn oil (control group; n _=_ 5). Blood collection and ultrasonographic assessment of the testes and supratesticular arteries (STAs) were conducted immediately before treatment (W0) and once weekly for six successive weeks after nano melatonin and melatonin injection (W1-W6). Results revealed decreases (*P* < 0.05) in the Doppler indices (resistive index; RI and pulsatility index; PI) of the testicular arteries at most time points of the study in the nano melatonin and melatonin groups. PIX of testicular parenchyma was significantly increased (P ˂ 0.05) in the treated groups compared to the control one. IND of testicular parenchyma increased significantly in the nano melatonin group compared to the melatonin and control groups. Testicular volume and scrotal circumference significantly increased (*P* < 0.05) in nano melatonin and melatonin groups compared to the control one. T concentration did not significantly (*P* > 0.05) change in the treated groups compared to the control group. E_2_, NO, and TAC concentrations increased (*P* < 0.05) in the treated groups compared to the control one. In conclusion, this study extrapolated that administrations of melatonin or nano melatonin can ameliorate the effects of environmental HS in prepubertal Ossimi ram lambs with a more protective effect and lower dose of nano melatonin.

## Introduction

Stress is triggered when there is an external stimulus that causes modifications to a biological system (Collier et al. 2019). In production animals, stress arises when an external event changes their health, basal metabolism, and productive capability (Al-Dawood 2017). When an animal is subjected to temperatures that exceed its physiological tolerance and capacity to adjust, heat stress (HS) occurs (Shahat et al. 2020). Sheep are homeothermic animals, which means they must maintain homeostasis by keeping their body temperature within a specific physiological range (Lu et al. 2019). Thus, HS could induce negative effects on the overall health status of sheep and decrease their productive and reproductive potential (Ribeiro et al. 2018). There are two ways that HS causes infertility in animals: either it affects the reproductive system directly through hyperthermia and the activation of the hypothalamic-pituitary-adrenal (HPA) axis, or it indirectly affects the body by decreasing feed intake, which lowers metabolic heat production and alters energy balance and nutrient availability (Aggarwal and Upadhyay 2013). Testicular blood flow (TBF) could be significantly affected if rams are exposed to HS circumstances. Hedia et al. (2019) reported decreased TBF during summer months compared to other months/year. Inadequate blood flow in the testis in heat-stressed rams resulted in testicular hypoxia and, in combination with direct hyperthermia, enhanced oxidative stress conditions through an increase in reactive oxygen species (ROS; Hamilton et al. 2016). ROS generation increases considerably during HS circumstances due to a decrease in superoxide dismutase (SOD) activity and SOD1 mRNA levels, which leads to an elevation of mitochondrial superoxide anion (SOA) formation. SOA reduces TBF via inactivating nitric oxide in the peroxynitrite production process, which changes endothelial-dependent vasodilation (Landmesser et al. 2003). So, the use of antioxidants could alleviate oxidative stress conditions in rams’ reproductive potentials.

Melatonin (N-acetyl-5-methoxytrypamine) is a neurohormone produced in the pineal gland as a primary site and in other sites such as the retina, skin, gastrointestinal cells, thymus, placenta, ovary, testicles, bone marrow, liver, and blood platelets (Reiter et al. 2010; Cebrián-Pérez et al. 2014). Melatonin has a significant impact on the regulation of reproductive functions in animals that are bred seasonally such as certain breeds of sheep and goats, by acting directly on the functional activity of the hypothalamic-pituitary-gonadal axis (Samir et al. 2020). Melatonin plays a pivotal antioxidant role at the cellular level, because of its ability to trap and neutralize free radicals through electron donation as a direct effect (Tan et al. 2007). However, melatonin has indirect effects by stimulating various antioxidant enzymes such as glutathione peroxidase/ reductase, catalase, and superoxide dismutase (Jang et al. 2010). Exogenous melatonin administration has been reported to boost ram testosterone secretion (Rekik et al. 2015) and increase testicular size (Rekik et al. 2015; Cevik et al. 2017). Melatonin increased TBF in Shiba goats (Samir et al. 2020) and Ossimi rams (El‐Shalofy et al. 2021).

Nanoparticles (NPs) show special functional features such as a specific surface area, multiple active surface centers, high surface activity, high catalytic efficacy, and potent adsorption capacity (Shi et al. 2010; Peters et al. 2004). Due to their physicochemical characteristics, nanoparticles can encapsulate a significant amount of drug, protecting it from degradation, and increasing its bioavailability, stability, and pharmacokinetic properties, while reducing possible toxic side effects (Martinelli et al. 2019). To create stable micelles or nano-micelles, an external supply of energy, such as heating or ultrasonication, is required (Pepić et al. 2014).

Testicular echogenicity (pixel intensity) variations correlate with testicular histomorphology (Giffin et al. 2009), testicular hemodynamics, and semen quality (Brito et al. 2012; Hedia et al. 2020a). Doppler ultrasound, however, is considered a good non-invasive imaging tool to assess testicular function in various animal species such as rams (Montes-Garrido et al. 2022), bulls (Gloria et al. 2018), and goats (Samir et al. 2020) through assessment of the blood flow dynamics of the testicular artery because it serves as the primary pathway for nutrition, oxygen, and other regulating hormones to and from the testis. This blood flow is critical for the extremely compact testis, where the seminiferous tubules are exposed to exceptionally low oxygen tension relative to the rest of the body tissues (Casao et al. 2010). Temperature variations in the testes and/or the surroundings can affect TBF; Setchell et al. 1995). Variations in TBF are frequently associated with significant alterations in systemic and seminal testosterone concentrations, as well as sperm production in rams (Batissaco et al. 2013; Hedia et al. 2020b), bulls (Claus et al. 2019), and stallions (Ortiz-Rodriguez et al. 2017).

Ossimi rams are one breed of Egyptian fat-tailed sheep farmed primarily for meat and wool production. This breed has limited reproductive potential during the summer months in Egypt (El-Shalofy et al. 2023). Numerous tools are available for assessing the reproductive performance of prepubertal rams such as scrotal circumference measurement, and hormone concentration analysis. Testosterone and estradiol are important biomarkers for assessing the reproductive capacity of rams because they strongly correlate with the scrotal circumference and play fundamental roles in spermatogenesis and sexual behavior (Belkadi et al. 2017). As far as we are aware, there hasn’t been any research on the impact of melatonin and nano melatonin on testicular hemodynamics along with testicular echotexture, scrotal circumference, concentration of testosterone, estradiol, nitric oxide (NO), and total antioxidant capacity (TAC) under environmental HS in prepubertal rams. We hypothesized that the administration of melatonin either in the commercial form or its nanoparticle form could alleviate heat stress conditions during summer months and improve ram reproductive performance. We aimed to ascertain the effect of a single s.c dose of melatonin and nano melatonin on testicular hemodynamics, echotexture, steroid production, NO, and antioxidant status in prepubertal Ossimi ram lambs under HS conditions.

## Materials and methods

This study was carried out on Ossimi ram lambs (Ovis aries), in the summer of 2023 at the educational farm, Faculty of Veterinary Medicine, Cairo University, Giza, Egypt. According to Egypt Meteorological Agency, the average temperature (T), relative humidity (RH), and temperature humidity index (THI) throughout the experimental period were 35.20 ± 0.60 °C, 59.60 ± 1.50%, and 87.05 ± 1.70, respectively. A previously published formula (El-Tarabany et al. 2017; Kendall and Webster 2009) was used to determine if the rams in this study were heat-stressed: THI = (1.8 × T + 32) − [(0.55 − 0.0055 × RH) × (1.8 × T − 26)]. This determined the three stress levels of the rams: not stressed (THI < 70), moderately stressed (THI = 70–80), and severely stressed (THI > 80). Consequently, the rams under examination (THI = 87.05 ± 1.70) were deemed to be under severe HS. All experimental procedures were accepted by the Ethics Committee for Animal Use at the Faculty of Veterinary Medicine, Cairo University, Egypt (approval number: Vet CU 25122023867).

### Preparation of melatonin nanoparticles

For the aqueous solution, melatonin was dissolved in a mixture of ethanol and water (1:1) solution. Polyvinyl alcohol (PVA) surfactant was added to the melatonin solution under magnetic stirring at 500 rpm for 1 h to obtain a homogenous stable aqueous phase. However, for the nano-emulsion preparation, the oil phase was prepared by taking the required volume of corn oil based on an optimized 1:10 ratio between oil and aqueous volumes. The melatonin-PVA solution and oil phase were mixed under magnetic stirring for 10 min to form a coarse emulsion (Silva et al. 2011).

#### Ultrasonication of nanoparticles

The coarse emulsion was further sonicated using an ultrasonic probe set at 60% amplitude and 1-second on-off pulse intervals. Total sonication time varied between 5 and 20 min to determine ideal parameters for smaller nano-emulsion droplet sizes (Ghosh et al. 2015).

#### Isolation and drying of prepared nanoparticles

The prepared melatonin nano-emulsion was centrifuged at 15,000 rpm to recover the precipitated nanoparticles followed by washing to remove residual oil and surfactants. Nanoparticles obtained were then lyophilized.

#### Characterization of nanoparticles

Melatonin nano-emulsion was studied physically and chemically to assess its biological potential. We examined the specimens microscopically for indexing, identification, and characterization with the aid of transmission electron microscopy (TEM, Jeol, JEM-2100 high-resolution, Japan). To determine the shape and surface topography of the melatonin nano-emulsions, an atomic force microscope (AFM, Agilent, USA) was used. A dynamic light scattering experiment was performed to determine how well particles dispersed in fluids based on their zeta potential and size.

**Fig. 1:**
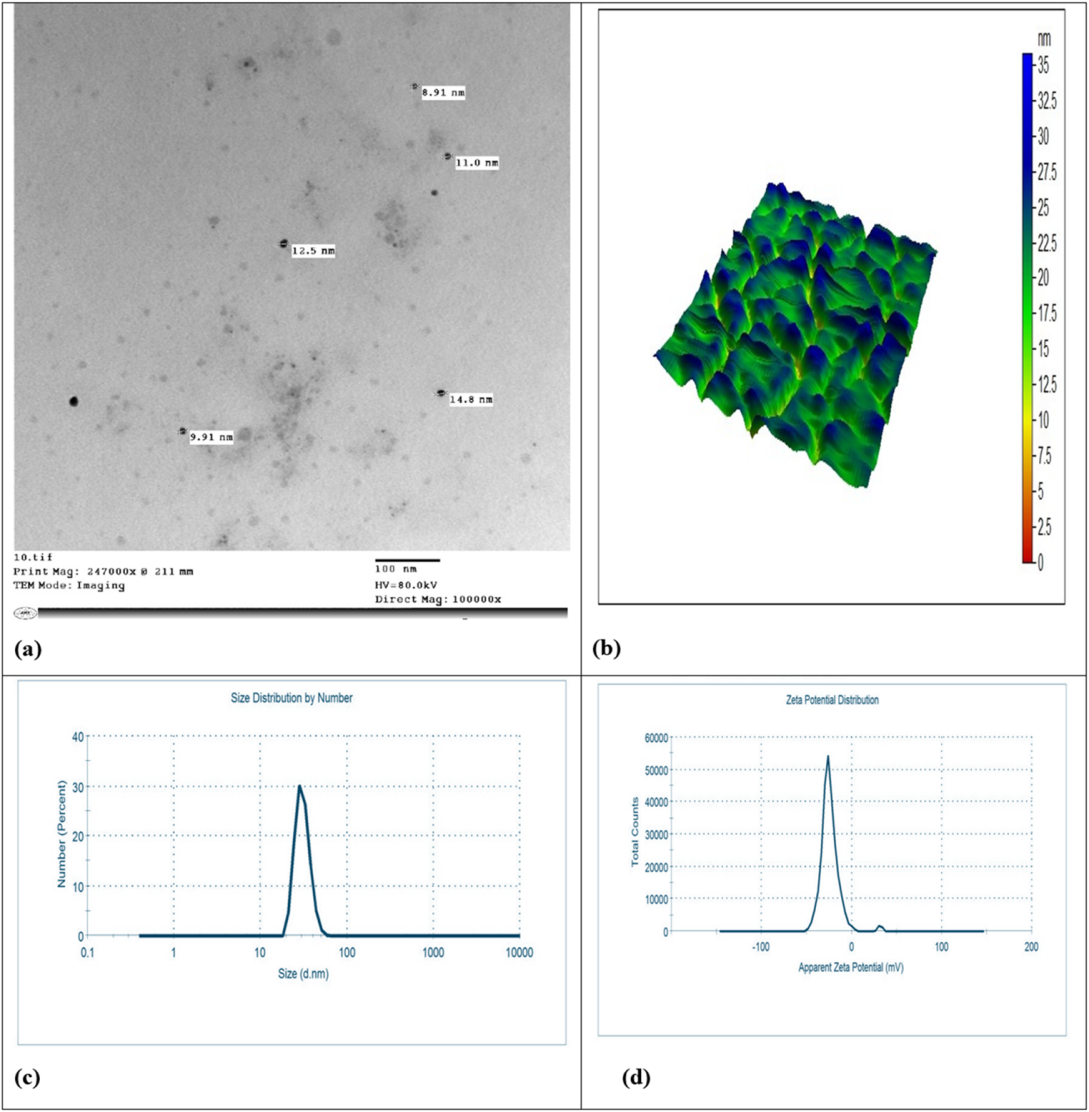
Characterization of melatonin nano-emulsion. With the aid of transmission electron microscopy, the particles in melatonin nano-emulsion were spherical and had a maximum thickness of 5–20 nm (**a**). Additionally, atomic force microscope images showed that melatonin nano-emulsion does not tend to aggregate in certain regions with homogeneously dispersed matrix (**b**). The results of zeta size showed that the average particle size of synthesized melatonin nano-emulsion is 18.2 ± 0.1 nm (**c**). Results of zeta potential showed 35.4 ± 0.05 mV as shown in **d**

Based on the TEM images shown in Fig. 1A, the particles in melatonin nano-emulsion were spherical and had a maximum thickness of 5–20 nm. Additionally, AFM images showed that melatonin nano-emulsion does not tend to aggregate in certain regions with homogeneous dispersed matrix (Fig. 1B). The results of zeta size showed that the average particle size of synthesized melatonin nano-emulsion is 18.2 ± 0.1 nm (Fig. 1C). Results of zeta potential showed 35.4 ± 0.05 mV as shown in Fig. 1D. Accordingly, the high zeta potential of the synthesized nano-emulsion directly impacts colloidal stability in water, as it derives from the high bioactivity of the melatonin nano-emulsion.

### Animals

**Fig. 2:**
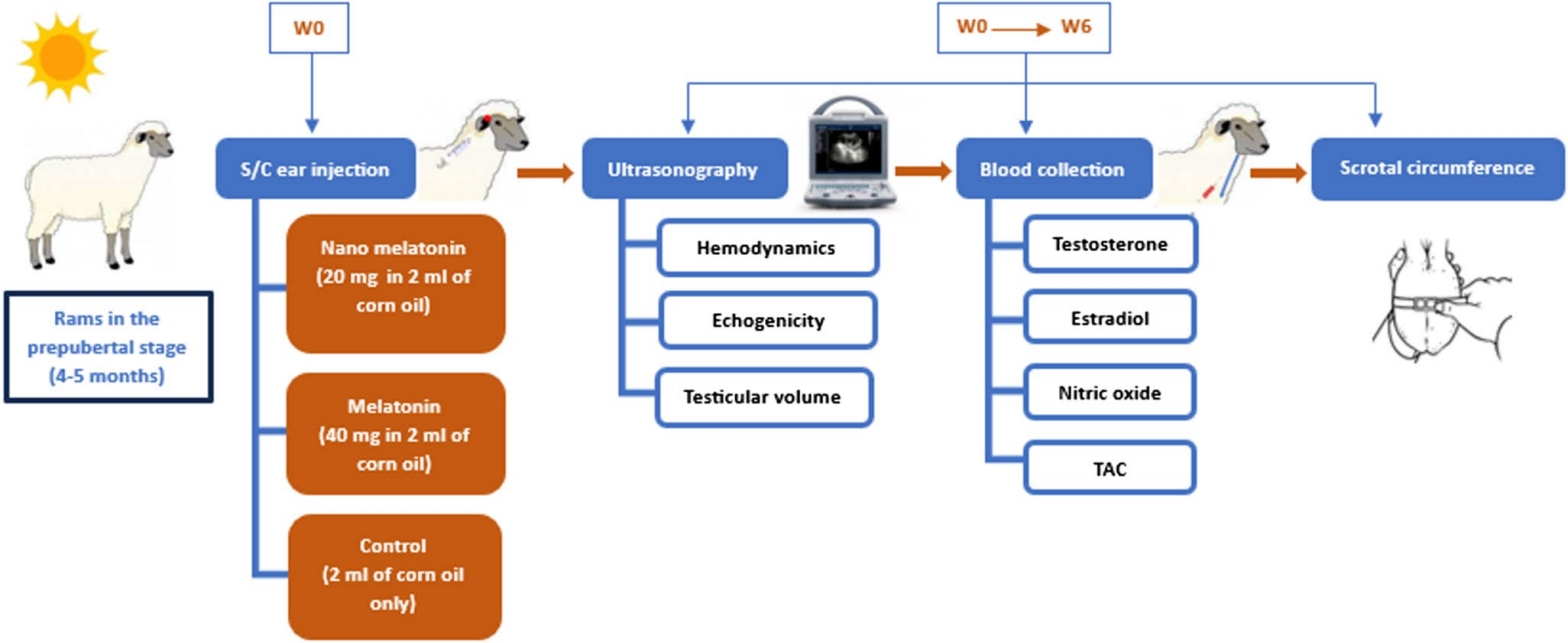
Schematic illustrations of the experimental procedures in this study

The experimental design is illustrated in Fig. 2. Fifteen ram lambs in the prepubertal stage, aged 4–5 months old (Osman 2010), and weighing 20–25 kg were used in the current study. Clinical and ultrasonographic examinations showed that they were clinically healthy and had no cardiovascular or reproductive problems. They were fed rations according to NRC recommendations and had *ad libitum* access to fresh water. They were routinely vaccinated and treated for external and internal parasites.

### Experimental design

Lambs were allocated into three equal groups (*n* = 5): (1) the nano melatonin group was treated with a single s.c dose of nano melatonin (20 mg/ram), (2) the melatonin group was treated with a single s.c dose of melatonin. Each ram lamb got 40 mg crystalline melatonin powder (Fujifilm Wako Pure Chemical Corporation, Osaka, Japan) dissolved in 2 ml of corn oil just before administration, and (3) control group which was injected with 2 ml of corn oil only. The dose of melatonin was selected based on a previous report (Samir et al. 2020). Ultrasound examination of the right and left testes, and blood sampling were performed on the day of injection (W0) and repeated a week for 6 consecutive weeks (W1-W6) after treatment.

### Ultrasonographic examinations of the testis of rams

Ultrasound examinations were performed by the same operator via a Doppler scanner equipped with a 7.5 MHz linear-array probe (EXAGO, IMV, France). The rams were gently confined without being sedated. Fine wool was shaved from both sides of the scrotum before every ultrasonographic test. A generous amount of scanning gel was used to guarantee contact and minimize scanning artifacts. All ultrasound machine settings (frequency, brightness, depth, and contrast) were standardized and fixed uniformly for all assessments.

Testicular hemodynamics in testicular arteries were monitored in this study. The testicular artery of rams seems convoluted right before entering the testis. The convoluted section is known as the supratesticular artery (STA). For pulsed-wave Doppler measurements, the transducer was positioned vertically on the side of the scrotum and then moved dorsally until the STA was visible within the vascular network at the proximal pole of the testis. After identifying the spectral layout of the right and left STAs, the resistance index (RI) and pulsatility index (PI) were measured. Blood perfusion of tissue downstream had an opposite relation with RI and PI readings of an artery (Araujo and Ginther 2009); as a result, when RI and PI increase, vascular resistance of blood flow increases, lowering blood perfusion, and vice versa (Samir et al. 2021). Two to four measurements were taken in points of interest along the STA to calculate each parameter (El-Shalofy and Hedia 2021). To evaluate the testicular echogenicity (pixel intensity), a clean, artifact-free picture of both testes was imaged by B-mode ultrasonography, frozen, and stored for later computed analysis. Using Adobe PhotoShop CC software, the stored pictures were retrieved and tested for testicular echogenicity by setting a 1 cm × 1 cm square in the testicular parenchyma in three distinct places regarding the central placement of the mediastinum testis (Giffin et al. 2009). Testicular parenchyma was examined for testicular length (L), width, (W), and thickness (T) using electronic calipers. Testicular volume (TV) was determined using the following formula, where TV = L × W × T × 0.61 (Montes-Garrido et al. 2023).

### Scrotal circumference

The scrotal circumference of the animals was measured (cm) at the greatest width of the testicle with a flexible measuring tape (Langford et al. 1989).

### Blood sampling

Blood samples (3 ml) were obtained from each lamb through jugular venipuncture and put into plain tubes just before ultrasonographic assessments. The taken blood samples were centrifuged for 10 min at 3000 rpm, after which the serum samples were collected and kept at -20 °C until additional hormonal and biochemical analyses were performed.

### Hormonal analysis

Concentrations of testosterone and estradiol were analyzed using the commercial ELISA kits (DiaSino Laboratories Co., Ltd. Zhengzhou, China) as described by the manufacturers. The intra- and inter-assay coefficients of variation, respectively, were 3.3 and 4.8% for testosterone and 3.8 and 5.6% for estradiol. Assay sensitivity was 0.05 ng/ml for testosterone and 20 pg/ml for estradiol.

### Biochemical analysis

Concentrations of NO and TAC were measured using commercial kits (Bio-diagnostic, Giza, Egypt) spectrophotometrically (Prietest TOUCH, Robonic, India) following the manufacturer’s instruction at a wavelength of 540 nm and 505 nm respectively. The NO intra-assay coefficient variation was 5.3%, with an assay sensitivity of 0.225 µmol/l in nitrite form. The TAC intra-assay coefficient was 3.4% and 0.04 mM/L sensitivity.

### Statistical analysis

The normality test was done on the data to determine its homogeneity and type, using the Kolmogorov-Smirnov test. Because there were no significant differences between the right and left testes of the rams studied, the data for each ram was combined and compared among the three groups. Data for Doppler and hormonal analysis were provided as means ± standard error of the mean (SEM). To investigate the effects of treatment as a fixed factor and time as a repeating factor, all parameters were evaluated for differences using repeated measures of two-way ANOVA. The Bonferroni post hoc test was used at different time points to assess the effect of treatment (3 levels: nano melatonin versus melatonin versus control) on changes in TBF in the STA, testicular echogenicity, testicular volume, scrotal circumference, T, E_2_, NO, and TAC concentrations. All statistical analyses were conducted using the GraphPad Prism5 program. *P* < 0.05 was used to indicate significance.

## Results

### Testicular hemodynamics

**Fig. 3:**
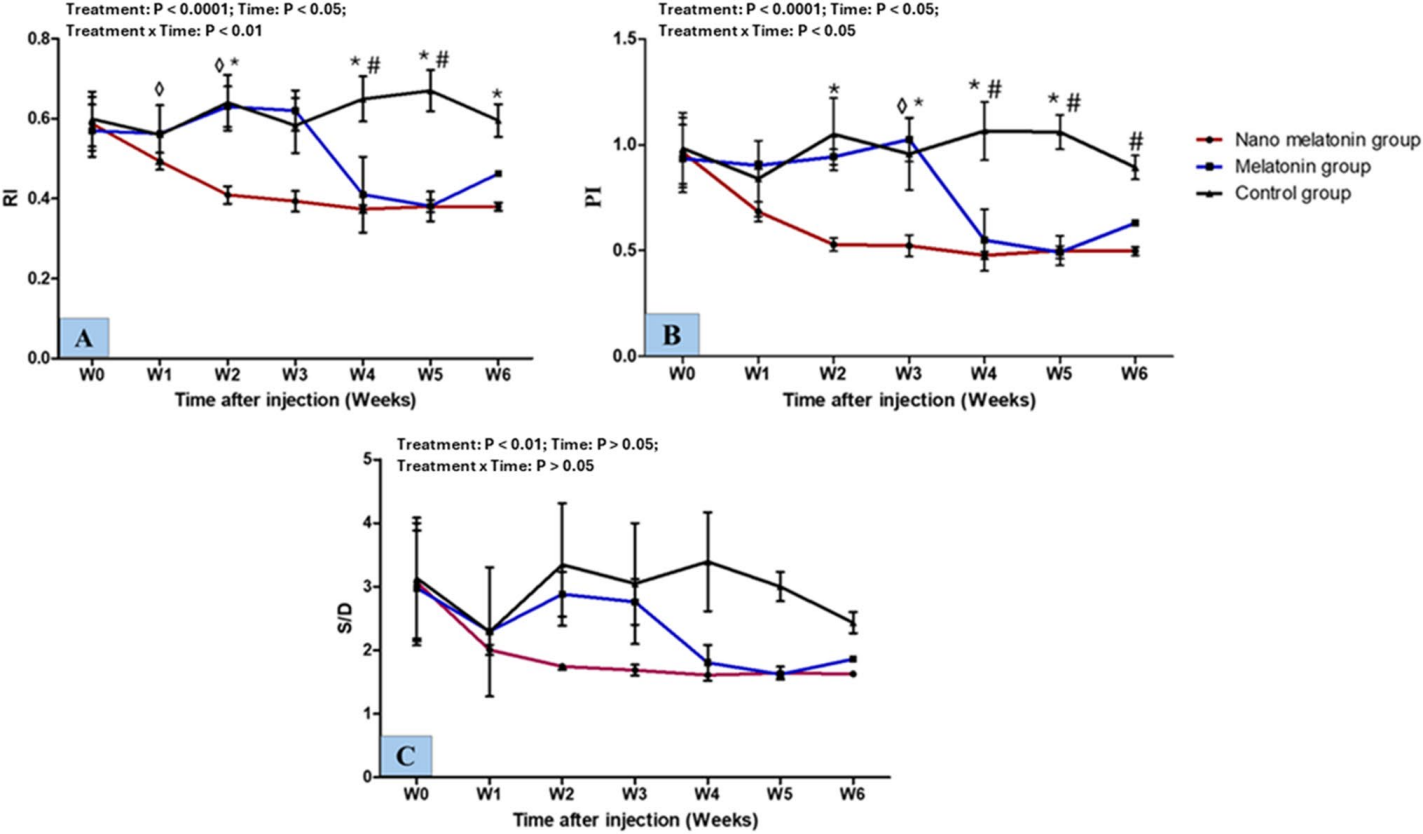
Changes in the parameters of testicular hemodynamics [resistive index; RI (**A**), pulsatility index; PI (**B**), and systolic/diastolic; S/D (**C**)] at the level of supratesticular artery as measured by pulsed-wave Doppler ultrasonography in prepubertal Ossimi ram lambs in the nano melatonin group, melatonin group, and the control group (*n* = 5, each) during different times (weeks). Values are means ± SEM. ◊ Values represent significant differences (*P* < 0.05) between nano melatonin and melatonin groups at the indicated times during the study. * Values represent significant (*P* < 0.05) differences between nano melatonin and control groups during the study. # Values represent significant (*P* < 0.05) differences between melatonin and control groups during the study

Referring to the experimental groups in this study, the changes in the parameters of testicular hemodynamics during the different times (weeks) are presented in Fig. 3. For the RI parameter, treatment, time, and interaction were significantly different (*P* < 0.0001, *P* < 0.05, and *P* < 0.01; respectively). RI values of blood flow were significantly lower (*P* < 0.05) in the nano melatonin group compared to the melatonin one. Compared to the control group, the nano melatonin group attained lower (*P* < 0.05) RI values. Furthermore, the melatonin group yielded lower (*P* < 0.05) RI values at W4, and W5 than the control one. Treatment, time, and interaction had notable differences (*P* < 0.0001, *P* < 0.05, and *P* < 0.05; respectively) in values of PI parameter. There were significant (*P* < 0.05) decreases in the values of PI in the nano melatonin group compared to the melatonin group and the control one. Lambs in the melatonin group had lower (*P* < 0.05) PI values than those in the control group. In general, there was a treatment effect (*P* < 0.01) in values of S/D among the groups.

### Testicular echogenicity

**Fig. 4:**
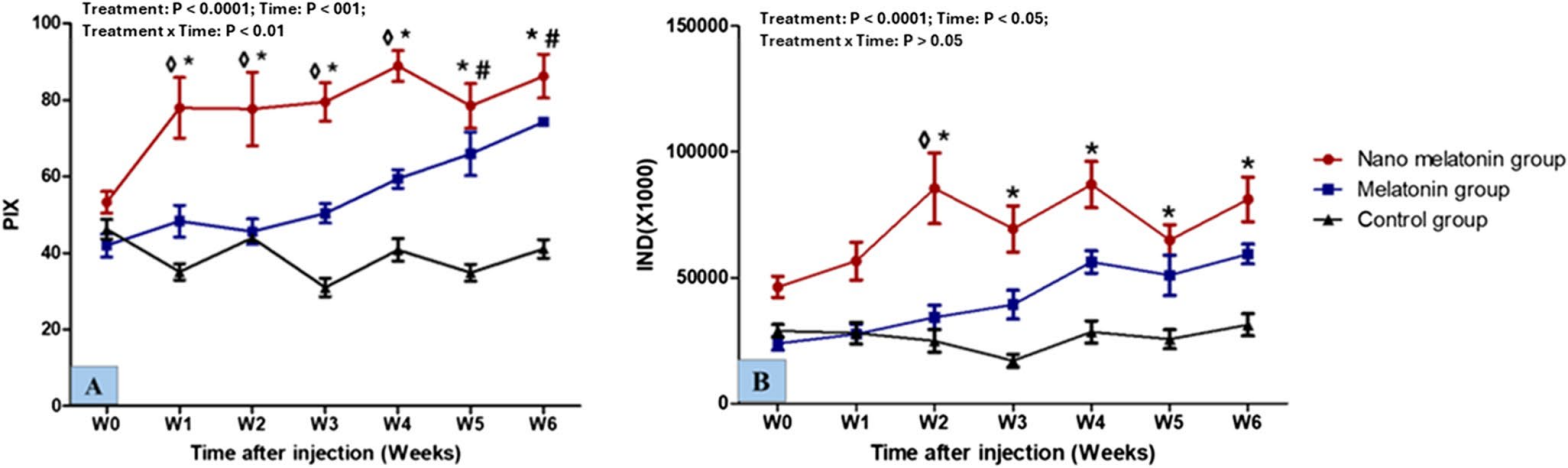
Changes in echotexture of testicular parenchyma [pixel intensity; PIX (**A**) and integrated density; IND (**B**)] as measured by computer analysis software in prepubertal Ossimi ram lambs in the nano melatonin group, melatonin group, and the control group (*n* = 5, each) during different times (weeks). Values are means ± SEM. ◊ Values represent significant differences (*P* < 0.05) between nano melatonin and melatonin groups at the indicated times during the study. * Values represent significant (*P* < 0.05) differences between nano melatonin and control groups during the study. # Values represent significant (*P* < 0.05) differences between melatonin and control groups during the study

The changes in echogenicity of the testicular parenchyma in the studied groups are shown in Fig. 4. In general, treatment, time, and interaction had notable differences (*P* < 0.0001, *P* < 001, and *P* < 0.01; respectively) in values of pixel intensity (PIX) testicular parenchyma. Lambs in the nano melatonin group had higher (*P* < 0.01) PIX values from W1 to W4 than those in the melatonin group. The nano melatonin group attained higher (*P* < 0.001) PIX values, compared to the control one. Values of PIX in the melatonin group were lower (*P* < 0.01), compared to that in the control one. Treatment and time were significantly different (*P* < 0.0001, and *P* < 0.05; respectively) in values of IND. There were significant (*P* < 0.001) increases in the values of IND of the testicular parenchyma in the nano melatonin group, compared to the melatonin group, and its values were higher (*P* < 0.05) compared to the control one. However, there were no significant (P ˃ 0.05) differences between melatonin and control groups in IND values.

### Testicular volume and scrotal circumference

**Fig. 5:**
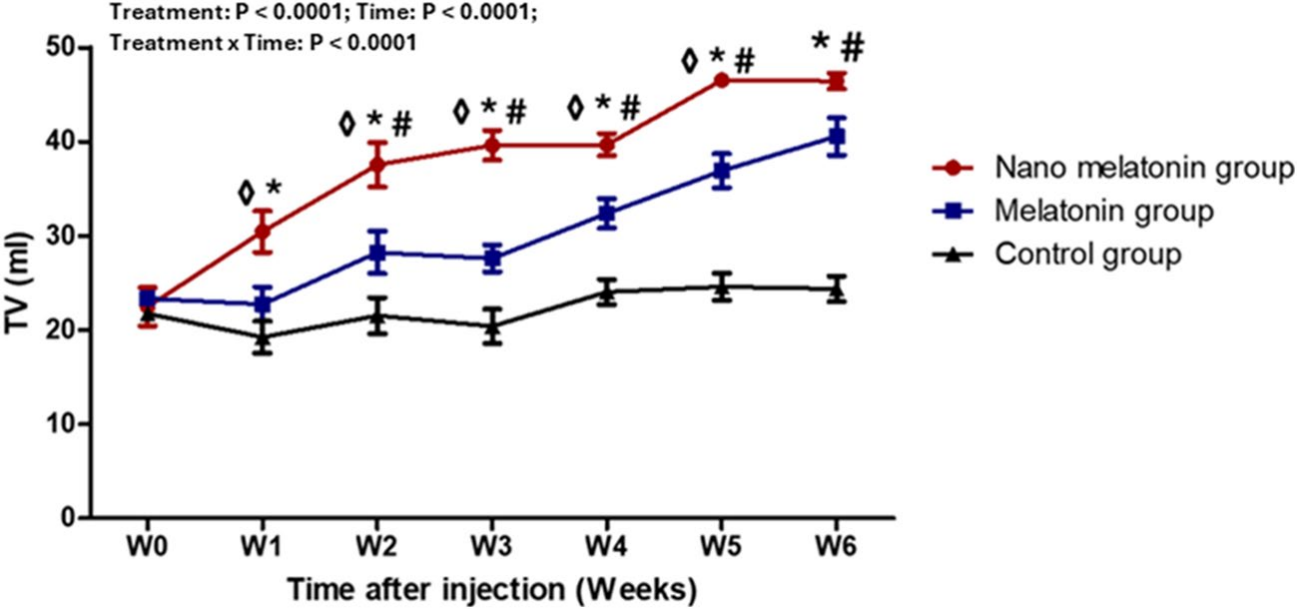
Changes in testicular volume (TV; ml) in prepubertal Ossimi ram lambs in the nano melatonin group, melatonin group, and the control group (*n* = 5, each) during different times (weeks). Values are means ± SEM. ◊ Values represent significant differences (*P* < 0.05) between nano melatonin and melatonin groups at the indicated times during the study. * Values represent significant (*P* < 0.05) differences between nano melatonin and control groups during the study. # Values represent significant (*P* < 0.05) differences between melatonin and control groups during the study

**Fig. 6:**
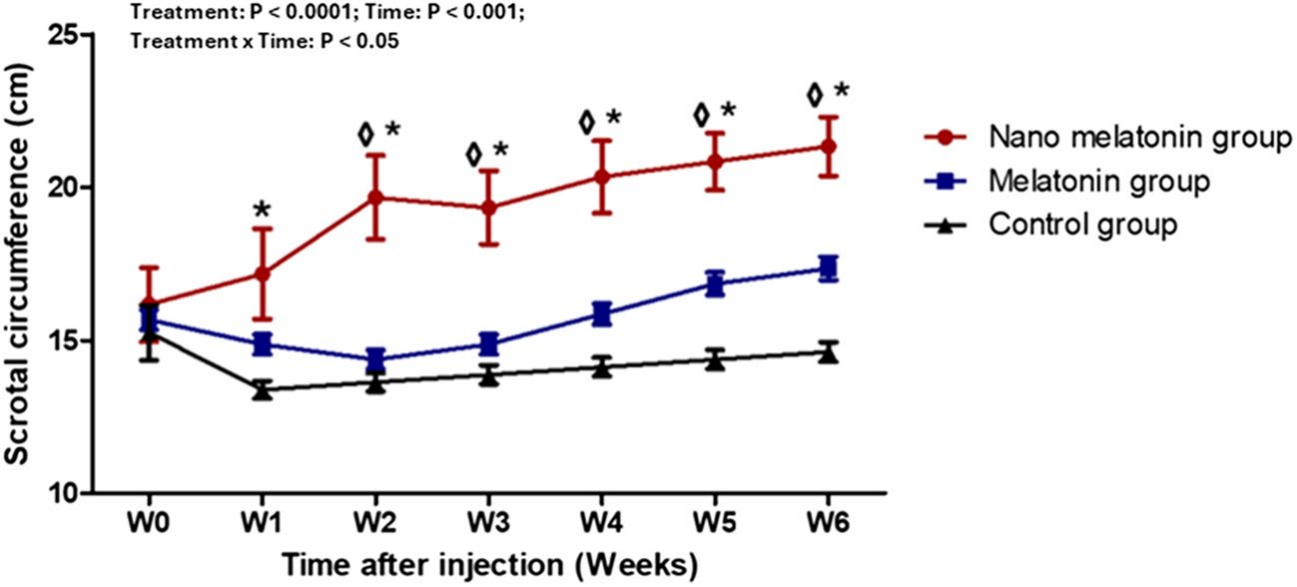
Changes in scrotal circumference (cm) in prepubertal Ossimi ram lambs in the nano melatonin group, melatonin group, and the control group (*n* = 5, each) during different times (weeks). Values are means ± SEM. ◊ Values represent significant differences (*P* < 0.05) between nano melatonin and melatonin groups at the indicated times during the study. * Values represent significant (*P* < 0.05) differences between nano melatonin and control groups during the study

During the study weeks, the effects of treatment, time, and interaction (*P* < 0.0001, for all) on testicular volume (ml) in rams were substantially observed (Fig. 5). In the nano melatonin group, testicular volume increased (P ˂ 0.05) significantly at the most time points of the study compared to melatonin and control groups. In melatonin-treated rams, testicular volume was greater (P ˂ 0.05) than the control group from study W2 and onwards. The effect of nano melatonin and melatonin administration on the scrotal circumference (cm) in the present study is presented in Fig. 6. In general, there were treatment, time, and interaction effects (*P* < 0.0001, *P* < 0.001, and *P* < 0.05; respectively) among groups. Increases in the scrotal circumference (*P* < 0.01) were observed in the nano melatonin group compared to that in the melatonin one and its levels were greater (*P* < 0.01) compared to that in the control group at most time points of the study.

### Hormonal and biochemical analysis

**Fig. 7:**
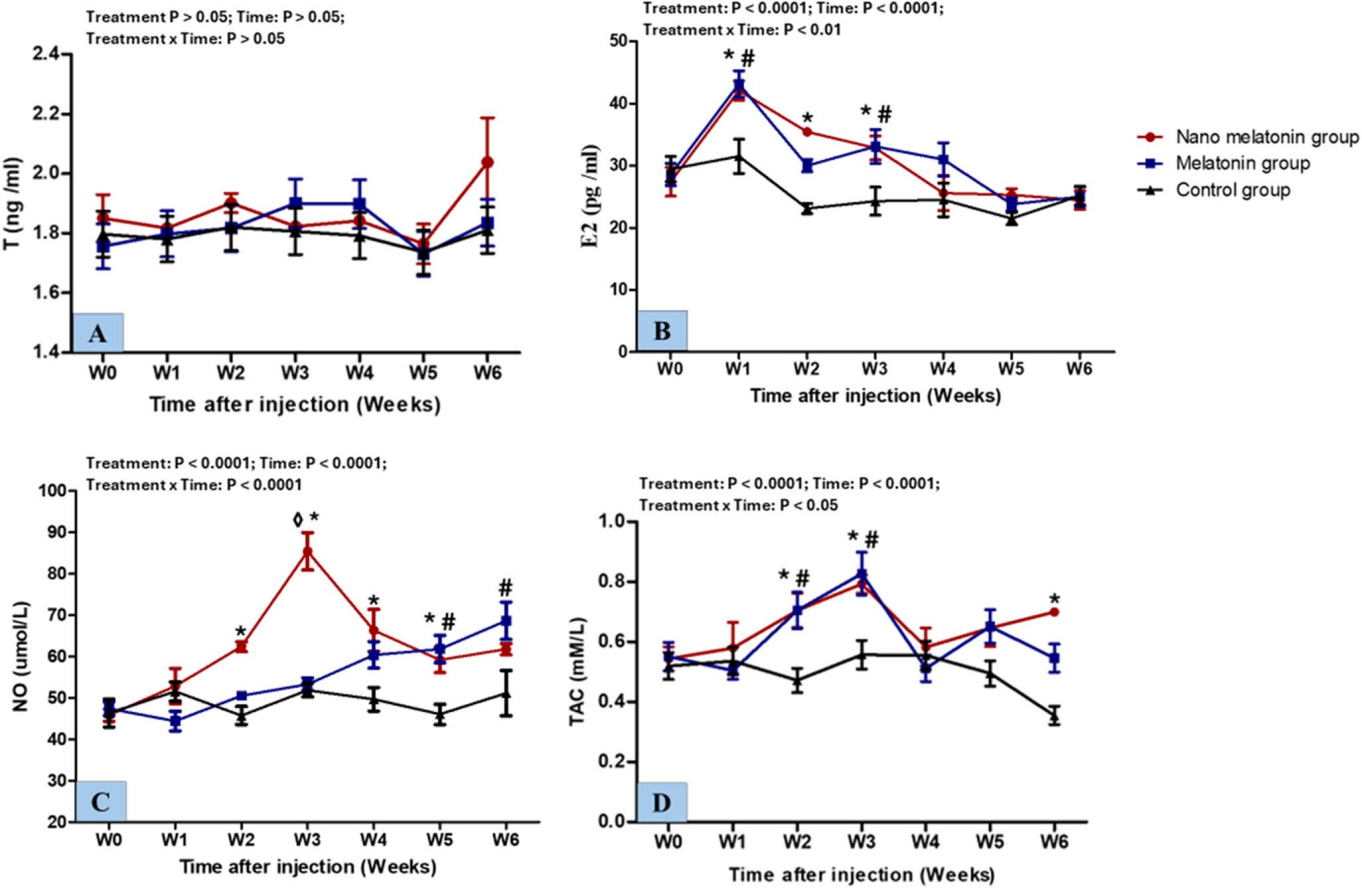
Changes in the concentrations of testosterone (T; ng/mL) (**A**), estradiol (E2; pg/mL) (**B**), nitric oxide (NO; µmol/L) (**C**), and total antioxidant capacity (TAC; Mm/L) (D) in prepubertal Ossimi ram lambs in the nano melatonin group, melatonin group, and the control group (*n* = 5, each) during different times (weeks). Values are means ± SEM. ◊ Values represent significant differences (*P* < 0.05) between nano melatonin and melatonin groups at the indicated times during the study. * Values represent significant (*P* < 0.05) differences between nano melatonin and control groups during the study. # Values represent significant (*P* < 0.05) differences between melatonin and control groups during the study

The serum levels of T (ng/ml), E_2_ (pg/ml), NO (µmol/L), and TAC ( mM/L) in the studied groups are shown in Fig. 7. Concentrations of T did not show a significant difference (*P* > 0.05) between the control and the treated groups during the study weeks. On the other hand, E_2_ concentration showed treatment, time, and interaction (*P* < 0.0001, *P* < 0.0001, and *P* < 0.01; respectively) effects. Compared to the control, the nano melatonin group attained higher (*P* < 0.05) E_2_ concentration. Furthermore, the melatonin group yielded a higher (*P* < 0.05) E_2_ concentration than the control one. However, there were no significant (*P* > 0.05) differences between the nano melatonin and melatonin groups in terms of E_2_ concentration during the study. For NO concentration, there were treatment, time, and interaction (*P* < 0.0001; for all) effects. Higher NO concentrations were found in the nano melatonin group in W3 compared to the melatonin group (P ˂ 0.001) and starting from W2 to W5 compared to the control one (*P* < 0.05). Moreover, concentrations of NO were significantly higher (*P* < 0.01) in the melatonin group compared to the control group. Regarding TAC concentration, treatment, time, and interaction were significantly different (*P* < 0.0001, *P* < 0.0001, and *P* < 0.05; respectively). Increases (*P* < 0.05) were noticed in the concentration of TAC in the nano melatonin group compared to the control group. Rams in the melatonin group attained higher (*P* < 0.05) serum TAC concentration than those in the control group.

## Discussion

Melatonin is commonly considered an antioxidant (Reiter et al. 2016; Galano and Reiter 2018) and it can be made more stable and soluble, and the duration of its pharmacological action can be extended, by encasing it in nanoparticles (Mirza-Aghazadeh-Attari et al. 2022). Thus, the current study sought to ascertain the beneficial effect of melatonin and, for the first time, nano melatonin on the TBF of prepubertal Ossimi ram lambs kept under environmental HS during the summer season. The findings of the current study support the hypothesis that a single dosage of melatonin and nano melatonin enhances testicular hemodynamics and echogenicity in prepubertal Ossimi ram lambs, particularly in hot and humid environments with better results in the nano melatonin group. The availability of this kind of data is essential for raising animal productivity and serving as a tool to help with the future solving of several issues of sheep fertility.

In this study, although administration of melatonin (40 mg/ram) decreased the Doppler indices (RI, PI, and S/D), lower doses of melatonin nanoparticles (20 mg/ram) caused more decrease in the Doppler indices in prepubertal Ossimi ram lambs. This was consistent with previous studies in which melatonin injections lowered RI and PI levels and enhanced TBF in rams (El-Shalofy et al. 2022; Egerszegi et al. 2014) and goats (Samir et al. 2020). Blood flow, defined as the total volume of blood passing through a spot, is influenced by two critical variables as it flows through a vessel: pressure and resistance. This relationship is represented as flow = pressure/resistance (Pinggera et al. 2008). Lowering RI and PI was followed by higher testicular functioning (steroidogenesis and spermatogenesis; Samir et al. 2015; Hedia et al. 2019). These enhancements were linked to melatonin’s systemic or local effects in the testis. Melatonin primarily regulates TBF through direct effects on the hypothalamus-pituitary-gonadal axis as observed in seasonally breeding mammals (Reiter et al. 2009). Additionally, melatonin has a vasodilatory effect that has been related to direct calcium channel blocking and indirect endothelium-dependent increases in NO and cGMP via MT2 melatonin receptor activation (Yuan et al. 2017; Zhao et al. 2017; Satake et al. 1991) that have been detected in the ram reproductive tract (González-Arto et al. 2017). The local impact of melatonin on testicular function may be triggered by the expression of melatonin receptors in testicular cells (Casao et al. 2012; Frungieri et al. 2017). Melatonin controls the transcription of aromatase, which is essential for the conversion of testosterone to estrogen (Odawara et al. 2009). Due to estrogen’s potent vasodilatory effect and its impact on testicular perfusion (Bollwein et al. 2008; Rosenfeld et al. 2002), the current study’s findings of significant increases in estrogen concentrations concurrent with decreases in RI and PI values of testicular arteries may highlight its potential role for regulating TBF.

The present study revealed that the administration of melatonin led to a substantial rise in the concentrations of TAC and NO in the melatonin and nano melatonin groups compared to the control one. Our results are consistent with Roshan et al. (2023) who reported that melatonin administration could elevate TAC in rams. Given that melatonin receptors have been found throughout the cardiovascular system, including endothelial vascular cells (Paulis and Šimko 2007), this finding may be related to the effect of melatonin as a potent antioxidant agent and free radical scavenger on the cardiovascular system (Cook et al. 2011). Indeed, melatonin has a critical antioxidant role that may be relevant in pathophysiological circumstances such as when oxidative stress is present (Malhotra et al. 2004; Samir et al. 2024). Likewise in mouse testicular injury caused by oxidative stress, melatonin enhanced TBF by protecting endothelial cells from reactive oxygen species (ROS; Yuan et al. 2017). Furthermore, it is important to note that ROS such as superoxide anion radical (O_2_^−^) quickly deactivates NO produced in vascular endothelial cells, producing peroxynitrite (Kissner et al. 1997). Antioxidants that are administered exogenously, such as melatonin, can efficiently scavenge ROS from the vasculature before their reaction with NO, increasing the bioavailability of NO (Thakor et al. 2010). So, elevated levels of NO, one of the most potent vasodilators, may have contributed to the stimulatory impact of melatonin on TBF that was seen in this experiment (Lissbrant et al. 1997; Paulis and Šimko 2007). Interestingly, the current study found stimulatory effects of nano melatonin on the studied parameters of TBF. In addition to the in vivo antioxidant effects of the prepared nanoparticles, encapsulation of melatonin in nanoparticles prevents degradation, enhances therapeutic properties, and reduces the required dose (Tursilli et al. 2006).

Assessment of the echotexture of testicular parenchyma is an additional noninvasive method for evaluating testicular function as reported in beef bulls (Brito et al. 2012). Testicular echogenicity significantly increased in the melatonin group in this study with obvious increases in the nano melatonin group than in the control one. Additionally, recent research suggested that the echogenic alterations of the testicular parenchyma are connected with increased levels of cellular condensation and vascular irrigation in rams (Hedia and El-Shalofy 2022). Increases in testicular echogenicity may be related to improvements in TAC, which may then ameliorate the status of oxidative stress and increase the functionality of the testis’s cellular matrix, which includes the spermatogenic, Leydig, and Sertoli cells (Camela et al. 2019).

In the current study, testicular volume and scrotal circumference increased considerably in the melatonin and nano-melatonin groups compared to the control group. Our findings were in agreement with those of Akar et al. (2023), who found that melatonin enhanced testicular volume, as well as those of Buffoni et al. (2015); Kleemann et al. (2021), who found that melatonin enhanced scrotal circumference in young rams. Testicular volume is primarily a reflection of spermatogenesis since the seminiferous tubules account for 70–80% of the testicular mass (Rehnberg 1993; Lin et al. 2009). Testicular size is frequently employed as a fertility indicator, and scrotal measurements serve as a base for selecting breeding rams (Toe et al. 2000) because testicular measures are highly heritable, easy to measure, and strongly linked with both spermatozoa production and semen quality (Rege et al. 2000).

The current study showed no significant differences in serum testosterone concentration between the treated and control groups. However, this non-significant difference in testosterone was likely sufficient to increase testicular volume and scrotal circumference in the treated groups. This result agrees with (Buffoni et al. 2015; Kleemann et al. 2022) but contradicted other studies that observed increasing circulating testosterone concentrations in melatonin groups. These differences could be due to variances in the developmental stage (El-Shalofy et al. 2022) as prepubertal rams may not yet have fully developed the hormonal pathways and receptors necessary for melatonin to influence testosterone production effectively, species (Samir et al. 2020), breed (Pool et al. 2020), Melatonin dosage (El-Shalofy et al. 2021), time of melatonin administration (Kleemann et al. 2021), method of melatonin administration (Kleemann et al. 2021), or duration of the study as the duration of the study might be insufficient to observe significant changes in testosterone levels.

## Conclusion

A single s.c dose of melatonin and nano melatonin improves TBF (as measured by color-pulsed Doppler ultrasonography), testicular echotexture, testicular volume, and scrotal circumference. Concomitantly there were increases in the concentrations of E_2_, TAC, and NO with a more significant effect and lower dose by nano melatonin in prepubertal Ossimi ram lambs under environmental heat stress conditions.

## Data Availability

The data that support this study are available in the article.

## References

[CR1] Aggarwal A, Upadhyay R (2013) Heat stress and animal productivity, vol 188. Springer, Delhi, India

[CR2] Akar M, Çevik M, Kocaman A (2023) Effects of melatonin administration on testicular volume, testicular hemodynamics, semen parameters, and antioxidant status during the non-breeding season in Bafra rams. SSRN 4431692 10.3390/ani14030442 PMC10854573 38338085

[CR3] Al-Dawood A (2017) Towards heat stress management in small ruminants review. Ann Anim Sci 17:59–88

[CR4] Araujo RR, Ginther OJ (2009) Vascular perfusion of reproductive organs in pony mares and heifers during sedation with detomidine or xylazine. Am J Vet Res 70:141–148 19119960 10.2460/ajvr.70.1.141

[CR5] Batissaco L, Celeghini ECC, Pinaffi FLV, de Olivera BMM, de Andrade AFC, Recalde ECS, Fernandes CB (2013) Correlations between testicular hemodynamic and sperm characteristics in rams. Braz J Vet Res Anim Sci 50:384–395

[CR6] Belkadi S, Safsaf B, Heleili N, Tlidjane M, Belkacem L, Oucheriah Y (2017) Seasonal influence on sperm parameters, scrotal measurements, and serum testosterone in Ouled Djellal breed rams in Algeria. Vet World 10(12):1486–1492. 10.14202/vetworld.2017.1486-1492 29391691 10.14202/vetworld.2017.1486-1492 PMC5771175

[CR7] Bollwein H, Schulze JJ, Miyamoto A, Sieme H (2008) Testicular blood flow and plasma concentrations of testosterone and total estrogen in the stallion after the administration of human chorionic gonadotropin. J Reprod Develop 54:335–339 10.1262/jrd.20014 18667792

[CR8] Brito LFC, Barth AD, Wilde RE, Kastelic JP (2012) Testicular ultrasonogram pixel intensity during sexual development and its relationship with semen quality, sperm production, and quantitative testicular histology in beef bulls. Theriogenology 78:69–76 22401830 10.1016/j.theriogenology.2012.01.022

[CR9] Buffoni A, Vozzi A, Gonzalez DM, Viegas H, LaTorraca A, Hozbor F, Abecia JA (2015) Melatonin modifies scrotal circumference but not plasma testosterone concentrations and semen quality of rams during the seasonal anestrus at 43 S. Biol Rhythm Res 46:785–795

[CR10] Camela ES, Nociti RP, Santos VJ, Macente BI, Murawski M, Vicente WR, Oliveira MEF (2019) Changes in testicular size, echotexture, and arterial blood flow associated with the attainment of puberty in Dorper rams raised in a subtropical climate. Reprod Domest Anim 54:131–137 29989218 10.1111/rda.13213

[CR11] Casao A, Vega S, Palacín I, Pérez-Pe R, Laviña A, Quintín FJ, Muiño‐Blanco T (2010) Effects of melatonin implants during non‐breeding season on sperm motility and reproductive parameters in Rasa Aragonesa rams. Reprod Domest Anim 45:425–432 18954380 10.1111/j.1439-0531.2008.01215.x

[CR12] Casao A, Gallego M, Abecia JA, Forcada F, Pérez-Pé R, Muino-Blanco T, Cebrián-Pérez JÁ (2012) Identification and immunolocalisation of melatonin MT1 and MT2 receptors in Rasa Aragonesa ram spermatozoa. Reprod Fertil Dev 24:953–961 22935156 10.1071/RD11242

[CR13] Cebrián-Pérez JA, Casao A, González‐Arto M, dos Santos Hamilton TR, Pérez‐Pé R, Muiño‐Blanco T (2014) Melatonin in sperm biology: breaking paradigms. Reprod Domest Anim 49:11–21 25277428 10.1111/rda.12378

[CR14] Cevik M, Yilmazer C, Kocyigit A (2017) Effects of melatonin implantation on the fertility potentials of Kivircik and Charollais ewes and rams during the non-breeding season. Pol J Vet Sci 20(3):501–506 29166263 10.1515/pjvs-2017-0060

[CR15] Claus LAM, Junior FAB, Junior CK, Pereira GR, da Cruz Fávaro P, Galdioli VHG, Seneda MM, de Azambuja Ribeiro EL (2019) Scrotal skin thickness, testicular shape and vascular perfusion using Doppler ultrasonography in bulls. Livest Sci 226:61–65

[CR16] Collier R, Baumgard LH, Zimbelman RB, Xiao Y (2019) Heat stress: physiology of acclimation and adaptation. Anim Front 9(1):12–19 32002234 10.1093/af/vfy031 PMC6951893

[CR17] Cook JS, Sauder CL, Ray CA (2011) Melatonin differentially affects vascular blood flow in humans. Am J Physiol Heart Circ Physiol 300(2):670–674 10.1152/ajpheart.00710.2010 PMC3044053 21148765

[CR18] Egerszegi I, Sarlós P, Rátky J, Solti L, Faigl V, Kulcsár M, Cseh S (2014) Effect of melatonin treatment on semen parameters and endocrine function in Black Racka rams out of the breeding season. Small Rumin Res 116(2–3):192–198

[CR19] El-Shalofy AS, Hedia MG (2021) Exogenous oxytocin administration improves the testicular blood flow in rams. Andrologia 53(10):14193 10.1111/and.14193 34309888

[CR20] El-Shalofy A, Hedia M, Kastelic J (2021) Melatonin improves testicular haemodynamics, echotexture and testosterone production in Ossimi rams during the breeding season. Reprod Domest Anim 56(11):1456–1463 34459033 10.1111/rda.14010

[CR21] El-Shalofy AS, Shahat AM, Hedia MG (2022) Effects of melatonin administration on testicular hemodynamics, echotexture, steroids production, and semen parameters during the non-breeding season in Ossimi rams. Theriogenology 184:34–40 35276486 10.1016/j.theriogenology.2022.02.027

[CR22] El-Shalofy AS, Samir H, El-Sherbiny HR (2023) Intramuscular administration of l-arginine boosts testicular hemodynamics, plasma concentrations of testosterone and nitric oxide in heat-stressed rams. Theriogenology 197:127–132 36502590 10.1016/j.theriogenology.2022.11.030

[CR23] El-Tarabany MS, El-Tarabany AA, Atta MA (2017) Physiological and lactation responses of Egyptian dairy Baladi goats to natural thermal stress under subtropical environmental conditions. Int J Biometeorol 61:61–68 27225437 10.1007/s00484-016-1191-2

[CR24] Frungieri MB, Calandra RS, Rossi SP (2017) Local actions of melatonin in somatic cells of the testis. Int J Mol Sci 18(6):1170 28561756 10.3390/ijms18061170 PMC5485994

[CR25] Galano A, Reiter RJ (2018) Melatonin and its metabolites vs oxidative stress: from individual actions to collective protection. J Pineal Res 65(1):12514 10.1111/jpi.12514 29888508

[CR26] Ghosh V, Mukherjee A, Chandrasekaran N (2015) Optimization of process parameters to develop nanoemulsion by ultrasound cavitation. Int J Pure Appl 37:33–38

[CR27] Giffin JL, Franks SE, Rodriguez-Sosa JR, Hahnel A, Bartlewski PM (2009) A study of morphological and haemodynamic determinants of testicular echotexture characteristics in the ram. Exp Biol Med 234(7):794–801 10.3181/0812-RM-364 19429851

[CR28] Gloria A, Carluccio A, Wegher L, Robbe D, Valorz C, Contri A (2018) Pulse wave doppler ultrasound of testicular arteries and their relationship with semen characteristics in healthy bulls. J Anim Sci Biotechnol 9(1):1–7 29441202 10.1186/s40104-017-0229-6 PMC5800041

[CR29] González-Arto M, Aguilar D, Gaspar-Torrubia E, Gallego M, Carvajal-Serna M, Herrera-Marcos LV, Casao A (2017) Melatonin MT1 and MT2 receptors in the ram reproductive tract. Int J Mol Sci 18(3):662 28335493 10.3390/ijms18030662 PMC5372674

[CR30] Hamilton TR, Mendes CM, Castro LS, Assis PM, Siqueira AF, Delgado JD, Goissis MD, Muiño-Blanco T, Cebrián-Pérez JÁ, Nichi M, Visintin JA (2016) Evaluation of lasting effects of heat stress on sperm profile and oxidative status of ram semen and epididymal sperm. Oxidative Med Cell Longev 2016(1):1687657 10.1155/2016/1687657 PMC4737001 26881013

[CR31] Hedia M, El-Shalofy A (2022) Ageing affects plasma steroid concentrations and testicular volume, echotexture and haemodynamics in rams. Andrologia 54(1):14309 10.1111/and.14309 34755370

[CR32] Hedia MG, El-Belely MS, Ismail ST, El-Maaty AMA (2019) Monthly changes in testicular blood flow dynamics and their association with testicular volume, plasma steroid hormones profile and semen characteristics in rams. Theriogenology 123:68–73 30292858 10.1016/j.theriogenology.2018.09.032

[CR33] Hedia MG, El-Belely MS, Ismail ST, El-Maaty AMA (2020a) Seasonal changes in testicular ultrasonogram pixel intensity and their association with semen characteristics in rams. Asian Pac J Reprod 9(1):49–54

[CR34] Hedia MG, El-Belely MS, Ismail ST, El‐Maaty AMA (2020b) Seasonal variation in testicular blood flow dynamics and their relation to systemic and testicular oxidant/antioxidant biomarkers and androgens in rams. Reprod Domest Anim 55(7):861–869 32374490 10.1111/rda.13696

[CR35] Jang HY, Kim YH, Kim BW, Park IC, Cheong HT, Kim JT, Yang BK (2010) Ameliorative effects of melatonin against hydrogen peroxide-induced oxidative stress on boar sperm characteristics and subsequent in vitro embryo development. Reprod Domest Anim 45(6):943–950 19473309 10.1111/j.1439-0531.2009.01466.x

[CR36] Kendall P, Webster J (2009) Season and physiological status affects the circadian body temperature rhythm of dairy cows. Livest Sci 125:155–160

[CR37] Kissner R, Nauser T, Bugnon P, Lye PG, Koppenol WH (1997) Formation and properties of peroxynitrite as studied by laser flash photolysis, high-pressure stopped-flow technique, and pulse radiolysis. Chem Res Toxicol 10(11):1285–1292 9403183 10.1021/tx970160x

[CR38] Kleemann DO, Kelly JM, Arney LJ, Len J, Tilbrook AJ, Walker SK (2021) Sexual behaviour, semen quality and fertility of young Border Leicester rams administered melatonin during spring. Anim Reprod Sci 231:106804 34271495 10.1016/j.anireprosci.2021.106804

[CR39] Kleemann DO, Kelly JM, Arney LJ, Tilbrook AJ, Walker SK (2022) Melatonin dose: testicular and testosterone response in Border Leicester rams during spring. Livest Sci 260:104928

[CR40] Landmesser U, Dikalov S, Price SR, McCann L, Fukai T, Holland SM, Mitch WE, Harrison DG (2003) Oxidation of tetrahydrobiopterin leads to uncoupling of endothelial cell nitric oxide synthase in hypertension. J Clin Invest 111(8):1201–1209 12697739 10.1172/JCI14172 PMC152929

[CR41] Langford GA, Shrestha JNB, Marcus GJ (1989) Repeatability of scrotal size and semen quality measurements in rams in a short-day light regime. Anim Reprod Sci 19(1–2):19–27

[CR42] Lin CC, Huang WJ, Chen KK (2009) Measurement of testicular volume in smaller testes: how accurate is the conventional orchidometer? J Androl 30(6):685–689 19578133 10.2164/jandrol.108.006460

[CR43] Lissbrant E, Löfmark U, Collin O, Bergh A (1997) Is nitric oxide involved in the regulation of the rat testicular vasculature? Biol Reprod 56(5):1221–1227 9160722 10.1095/biolreprod56.5.1221

[CR44] Lu Z, Ma Y, Li Q, Liu E, Jin M, Zhang L, Wei C (2019) The role of N 6-methyladenosine RNA methylation in the heat stress response of sheep (Ovis aries). Cell Stress Chaperones 24:333–342 30701478 10.1007/s12192-018-00965-x PMC6439051

[CR45] Malhotra S, Sawhney G, Pandhi P (2004) The therapeutic potential of melatonin: a review of the science. Medscape Gen Med 6(2):46 PMC1395802 15266271

[CR46] Martinelli C, Pucci C, Ciofani G (2019) Nanostructured carriers as innovative tools for cancer diagnosis and therapy. APL Bioeng 3(1):011502 31069332 10.1063/1.5079943 PMC6481740

[CR47] Mirza-Aghazadeh-Attari M, Mihanfar A, Yousefi B, Majidinia M (2022) Nanotechnology-based advances in the efficient delivery of melatonin. Cancer Cell Int 22(1):43 35093076 10.1186/s12935-022-02472-7 PMC8800219

[CR48] Montes-Garrido R, Riesco MF, Anel-Lopez L, Neila-Montero M, Palacin-Martinez C, Boixo JC, Alvarez M (2022) Application of ultrasound technique to evaluate the testicular function and its correlation to the sperm quality after different collection frequency in rams. Front Vet Sci 9:1035036 36504850 10.3389/fvets.2022.1035036 PMC9732105

[CR49] Montes-Garrido R, Anel-Lopez L, Riesco MF, Neila-Montero M, Palacin-Martinez C, Soriano-Úbeda C, Alvarez M (2023) Does size matter? Testicular volume and its predictive ability of sperm production in rams. Animals 13(20):3204 37893928 10.3390/ani13203204 PMC10603633

[CR50] Odawara H, Iwasaki T, Horiguchi J, Rokutanda N, Hirooka K, Miyazaki W, Koibuchi N (2009) Activation of aromatase expression by retinoic acid receptor-related orphan receptor (ROR) α in breast cancer cells: identification of a novel ROR response element. J Biol Chem 284(26):17711–17719 19439415 10.1074/jbc.M109.009241 PMC2719410

[CR51] Ortiz-Rodriguez JM, Anel-Lopez L, Martín-Muñoz P, Álvarez M, Gaitskell-Phillips G, Anel L, Ortega Ferrusola C (2017) Pulse doppler ultrasound as a tool for the diagnosis of chronic testicular dysfunction in stallions. PLoS ONE 12(5):0175878 10.1371/journal.pone.0175878 PMC5448730 28558006

[CR52] Osman AM (2010) Gonadal and epididymal sperm counts of growing Ossimi rams in upper Egypt. Assiut Vet Med J 56(125):1–11. 10.21608/avmj.2010.173948

[CR53] Paulis L, Šimko F (2007) Blood pressure modulation and cardiovascular protection by melatonin: potential mechanisms behind. Physiol Res 56(6):671–684 18197748 10.33549/physiolres.931236

[CR54] Pepić I, Lovrić J, Hafner A, Filipović-Grčić J (2014) Powder form and stability of pluronic mixed micelle dispersions for drug delivery applications. Drug Dev Ind Pharm 40(7):944–951 23627442 10.3109/03639045.2013.791831

[CR55] Peters K, Unger RE, Kirkpatrick CJ, Gatti AM, Monari E (2004) Effects of nano-scaled particles on endothelial cell function in vitro: studies on viability, proliferation and inflammation. J Mater Sci: Mater Med 15:321–325 15332593 10.1023/b:jmsm.0000021095.36878.1b

[CR56] Pinggera GM, Mitterberger M, Bartsch G, Strasser H, Gradl J, Aigner F, Frauscher F (2008) Assessment of the intratesticular resistive index by colour Doppler ultrasonography measurements as a predictor of spermatogenesis. BJU Int 101(6):722–726 18190642 10.1111/j.1464-410X.2007.07343.x

[CR57] Pool KR, Rickard JP, Pini T, De Graaf SP (2020) Exogenous melatonin advances the ram breeding season and increases testicular function. Sci Rep 10(1):9711 32546776 10.1038/s41598-020-66594-6 PMC7297710

[CR58] Rege JEO, Toe F, Mukasa-Mugerwa E, Tembely S, Anindo D, Baker RL, Lahlou-Kassi A (2000) Reproductive characteristics of Ethiopian Highland sheep: II. Genetic parameters of semen characteristics and their relationships with testicular measurements in ram lambs. Small Rumin Res 37(3):173–187 10867315 10.1016/s0921-4488(00)00140-1

[CR59] Rehnberg GL (1993) Collection of interstitial fluid and seminiferous tubule fluid from the rat testis. Methods Toxicol 3:265–273

[CR60] Reiter RJ, Tan DX, Manchester LC, Paredes SD, Mayo JC, Sainz RM (2009) Melatonin and reproduction revisited. Biol Reprod 81(3):445–456 19439728 10.1095/biolreprod.108.075655

[CR61] Reiter RJ, Tan DX, Fuentes-Broto L (2010) Melatonin: a multitasking molecule. Prog Brain Res 181:127–151 20478436 10.1016/S0079-6123(08)81008-4

[CR62] Reiter RJ, Mayo JC, Tan DX, Sainz RM, Alatorre-Jimenez M, Qin L (2016) Melatonin as an antioxidant: under promises but over delivers. J Pineal Res 61(3):253–278 27500468 10.1111/jpi.12360

[CR63] Rekik M, Taboubi R, Ben Salem I, Fehri Y, Sakly C, Lassoued N, Hilali ME (2015) Melatonin administration enhances the reproductive capacity of young rams under a southern Mediterranean environment. J Anim Sci 86(7):666–672 10.1111/asj.12350 25689168

[CR64] Ribeiro BP, Lanferdini E, Palencia JY, Lemes MA, de Abreu ML, de Souza Cantarelli V, Ferreira RA (2018) Heat negatively affects lactating swine: a meta-analysis. J Therm Biol 74:325–330 29801645 10.1016/j.jtherbio.2018.04.015

[CR65] Rosenfeld CR, Roy T, Cox BE (2002) Mechanisms modulating estrogen-induced uterine vasodilation. Vasc Pharmacol 38(2):115–125 10.1016/s0306-3623(02)00135-0 12379958

[CR66] Roshan NJ, Garoussi MT, Akbarinejad V (2023) Evaluation of the effect of melatonin implantation in rams and eCG dose in ewes synchronized by a CIDR-eCG protocol on reproductive performance of Lacaune sheep breed during non-breeding season. Anim Reprod Sci 259:107365 37980808 10.1016/j.anireprosci.2023.107365

[CR67] Samir H, Sasaki K, Ahmed E, Karen A, Nagaoka K, El Sayed M, Watanabe G (2015) Effect of a single injection of gonadotropin-releasing hormone (GnRH) and human chorionic gonadotropin (hCG) on testicular blood flow measured by color doppler ultrasonography in male Shiba goats. J Vet Med Sci 77(5):549–556 25715956 10.1292/jvms.14-0633 PMC4478734

[CR68] Samir H, Nyametease P, Elbadawy M, Nagaoka K, Sasaki K, Watanabe G (2020) Administration of melatonin improves testicular blood flow, circulating hormones, and semen quality in Shiba goats. Theriogenology 146:111–119 32078960 10.1016/j.theriogenology.2020.01.053

[CR69] Samir H, Radwan F, Watanabe G (2021) Advances in applications of color Doppler ultrasonography in the andrological assessment of domestic animals: a review. Theriogenology 161:252e61 33341504 10.1016/j.theriogenology.2020.12.002

[CR70] Samir H, Elfadadny A, Radwan F, El-Sherbiny HR, Swelum AA, Khalil WA, Watanabe G (2024) Spatial local expressions of kisspeptin in the uterus and uterine tubes and its relationship to the reproductive potential in goats. Domest Anim Endocrinol 88:106850. 10.1016/j.domaniend.2024.106850 38640803 10.1016/j.domaniend.2024.106850

[CR71] Satake N, Oe H, Shibata S (1991) Vasorelaxing action of melatonin in rat isolated aorta; possible endothelium-dependent relaxation. Gen Pharmacol 22(6):1127–1133 1667303 10.1016/0306-3623(91)90589-x

[CR72] Setchell BP, Bergh A, Widmark A, Damber JE (1995) Effect of testicular temperature on vasomotion and blood flow. Int J Androl 18(3):120–126 7558374 10.1111/j.1365-2605.1995.tb00397.x

[CR73] Shahat AM, Rizzoto G, Kastelic JP (2020) Amelioration of heat stress-induced damage to testes and sperm quality. Theriogenology 158:84–96 32947064 10.1016/j.theriogenology.2020.08.034

[CR74] Shi LG, Yang RJ, Yue WB, Xun WJ, Zhang CX, Ren YS, Lei FL (2010) Effect of elemental nano-selenium on semen quality, glutathione peroxidase activity, and testis ultrastructure in male Boer goats. Anim Reprod Sci 118(2–4):248–254 19914014 10.1016/j.anireprosci.2009.10.003

[CR75] Silva SV, Soares AT, Batista AM, Almeida FC, Nunes JF, Peixoto CA, Guerra MMP (2011) In vitro and in vivo evaluation of ram sperm frozen in tris egg-yolk and supplemented with superoxide dismutase and reduced glutathione. Reprod Domest Anim 46:874–881 21332830 10.1111/j.1439-0531.2011.01758.x

[CR76] Tan DX, Manchester LC, Terron MP, Flores LJ, Reiter RJ (2007) One molecule, many derivatives: a never-ending interaction of melatonin with reactive oxygen and nitrogen species? J Pineal Res 42(1):28–42 17198536 10.1111/j.1600-079X.2006.00407.x

[CR77] Thakor AS, Herrera EA, Serón-Ferré M, Giussani DA (2010) Melatonin and vitamin C increase umbilical blood flow via nitric oxide‐dependent mechanisms. J Pineal Res 49(4):399–406 20958954 10.1111/j.1600-079X.2010.00813.x

[CR78] Toe F, Rege JEO, Mukasa-Mugerwa E, Tembely S, Anindo D, Baker RL, Lahlou-Kassi A (2000) Reproductive characteristics of Ethiopian Highland sheep: I. Genetic parameters of testicular measurements in ram lambs and relationship with age at puberty in ewe lambs. Small Rumin Res 36(3):227–240 10781739 10.1016/s0921-4488(99)00117-0

[CR79] Tursilli R, Casolari A, Iannuccelli V, Scalia S (2006) Enhancement of melatonin photostability by encapsulation in lipospheres. J Pharm Biomed Anal 40(4):910–914 16242283 10.1016/j.jpba.2005.08.025

[CR80] Yuan XC, Wang P, Li HW, Wu QB, Zhang XY, Li BW, Xiu RJ (2017) Effects of melatonin on spinal cord injury-induced oxidative damage in mice testis. Andrologia 49(7):12692 10.1111/and.12692 27595881

[CR81] Zhao T, Zhang H, Jin C, Qiu F, Wu Y, Shi L (2017) Melatonin mediates vasodilation through both direct and indirect activation of BKCa. Channels J Mol Endocrinol 59(3):219–233 28676563 10.1530/JME-17-0028

